# Blinking characterization from high speed video records. Application to biometric authentication

**DOI:** 10.1371/journal.pone.0196125

**Published:** 2018-05-07

**Authors:** Julián Espinosa, Begoña Domenech, Carmen Vázquez, Jorge Pérez, David Mas

**Affiliations:** 1 Department of Optics, Pharmacology and Anatomy, University of Alicante, Alicante, Spain; 2 University Institute of Physics Applied to Sciences and Technologies, University of Alicante, Alicante, Spain; Tokai University, JAPAN

## Abstract

The evaluation of eye blinking has been used for the diagnosis of neurological disorders and fatigue. Despite the extensive literature, no objective method has been found to analyze its kinematic and dynamic behavior. A non-contact technique based on the high-speed recording of the light reflected by the eyelid in the blinking process and the off-line processing of the sequence is presented. It allows for objectively determining the start and end of a blink, besides obtaining different physical magnitudes: position, speed, eyelid acceleration as well as the power, work and mechanical impulse developed by the muscles involved in the physiological process. The parameters derived from these magnitudes provide a unique set of features that can be used to biometric authentication. This possibility has been tested with a limited number of subjects with a correct identification rate of up to 99.7%, thus showing the potential application of the method.

## Introduction

Eye blinking is one of the fastest human reflexes [1]. A blink is a temporary closure of both eyes involving movements of upper and lower eyelids. Blinks’ role is fundamentally keeping the eye hydrated, allowing the tear film distribution over the ocular surface [2,3], and protecting against foreign objects [4,5]. It represents a normal, simply observable and easily accessible phenomenon that reflects central nervous activation processes without voluntary manipulation. Eyelids movements require simple neural commands and few active forces, so their analysis may reveal any abnormality and show if it is derived from a muscular or a neural disorder [4,6]. In all types of blinks, i.e. spontaneous, reflex and voluntary blinks, the movement of the upper eyelid is a result of three active forces (the orbicularis oculi -OO- muscle, the levator palpebrae -LP- muscle and Mueller’s muscle) and a passive force produced by the mechanical arrangement of the eyelid [4,7]. The tonic activity of the LP holds the upper eyelid against passive downward forces. The eyelid drops due to the inhibition of the LP and the activation of the OO muscles. Then, it opens again when the OO muscle activity has turned off and the LP has returned to its tonic activity.

In the past four decades, there have been a huge number of longitudinal studies involving the eye blink. Environmental conditions, age and gender variations in blink rate have been reported [8–11]. In healthy subjects, blinking frequency decrease when subjects are conducting tasks with high cognitive and visual demands [12,13]. Esteban [14] asserted the blink reflex evaluation as an essential tool for the diagnosis and pathophysiological insight into an important number of human neurological disorders. The spontaneous eye blink is also considered a suitable ocular indicator for fatigue diagnostics [15,16] and drowsiness measurement [17,18]. On the other hand, Shultz et al. [19] showed that measures of blink inhibition timing can serve as precise markers of perceived stimulus salience and they are useful quantifiers of atypical processing of social affective signals in toddlers. Another recent application is human biometric for authentication purposes. Abo-Zahhad et al. [20] achieved a high recognition rate (up to 97.3%) from blink waveforms of 25 subjects extracted from electro-oculograms (EOG) signals.

Traditionally, the eye blink was assessed by procedures requiring the application of electrodes to monitor the OO electromyographic activity and get the EOG signal [20–26], or the use of direct magnetic search coil technique [27–30]. Nevertheless, contact-free recording procedures such as photo or video, that permit a quantitative assessment for eye movement during blinking without interfering with the subject, have also been used [2,3,13,17,18,31–47].

The most evaluated blink properties are the rate and the duration because of their relationship with mental stages as fatigue, lapses of attention and stress. The start and the end of the blink are usually considered interdependent and they are determined through the definition of pre-calibrated threshold variables. Indeed, an objective method to determine the end of a blink has not been reported [30]. Other blink features like amplitudes and speeds are also assessed in literature but, up to our knowledge, a thorough report that gathers and analyzes all the physical magnitudes related to the kinematics and dynamics of the process has not been yet published.

An accurate evaluation of the blinking process through video recording needs of high-speed videos. In normal speed videos (60 fps), the difference in the position of the eyelid between two frames may be too large to track it precisely. Hence, Bernard et al. [31], with an eye tracking system, and Corthout et al. [32], with a high-speed Kodak camera, video monitored eye blinks with a temporal resolution of 2 ms.

Some years ago, some of the authors of this work presented a non-invasive technique aimed to high speed measure some of the blinking dynamic features, with 2 ms of temporal resolution too [33]. Lid displacement was monitored by studying the saturation of the frames in the sequence that allowed a quantitative analysis of eyelid location at any instant. In a posterior work [48], the authors proposed an analytical model of eye blinking including lid movement and ocular retraction.

In this paper, we have refined the technique and the blink action is thoroughly described from the analysis of different physical magnitudes directly related to the muscles action. In a recorded sequence of a subject blinking, the eyelid position is directly related to changes in the reflected light. From the variation of the position in time, its first and second derivatives, and their product, we have obtained a set of features describing this physiological phenomenon. As result, the obtained average values of some of the eye blink features agree with those reported in the literature [6,44,45]. Others related to muscular dynamics (power, work and impulse) are reported here for the first time.

Technological advances in last years have enabled the development of new biometric identification systems [20, 49, 50] based on human physical or physiological characteristics that can be studied using digital image processing. Therefore, among the wide field of applications where the analysis of the blink biomechanics could be of interest, we have evaluated the performance of the extracted blink features to accurately authenticate subjects through biometrics.

The paper is structured as follows. In the next section, we describe the subjects that participated in the experiment, the experimental setup and the method used to characterize the blinking. We define different physical features related to the blinking kinematics and dynamics. The third section deals with average results of the blinking of the subjects under study. There, we also introduce an application of the procedure to biometric authentication using different classification algorithms and sets of blinking data. The proposal is tested on a reduced number of subjects in order to check its viability. Finally, in the discussion and conclusion section, we discuss pros and contra of using the method to biometric authentication and present the main conclusions.

### Subjects and methods

Our method was tested on 26 subjects (13 females and 13 males of ages ranging from 21 to 62 years, 38±14). Students and staff from the department were recruited as participants without compensation. No subject was discarded from the study. Video sequences were recorded using a commercial camera (GOPRO HERO 3+) working at 240 frames per second. We adhered to the tenets of the Declaration of Helsinki during this study. All participants were informed about the nature and purpose of the study and all of them provided written informed consent. Experiments were conducted in winter of 2016 with the approval of the "Comité de Ética de la Universidad de Alicante. Nº Expediente UA-2016-04-11". Subjects rested their head on a chinrest and the camera was placed in front of their faces at a distance of 30 cm. A halogen lamp was used to illuminate the scene. Subjects were asked to blink naturally during each sequence, which lasted 20 seconds. Seven sequences per subject were recorded.

An image processing algorithm based on the difference in light reflection between the eyelid and the open eye (the pupil, the iris and the sclera) [38] has been implemented. Visible light, as well as infrared radiation, is considerably more absorbed by the pupil and the iris than it is by the eyelid [43]. Some of the authors of this work showed that the variation in the intensity mean value of the blinking image provides direct information about the eyelid position [33]. Thus, by selecting the appropriate region of interest (ROI) around eye, one can observe that the energy dispersed is a direct function of the closure status of the eye. This way Lee et al. [39] obtained the cumulative difference of the number of black pixels of an eye region using an adaptive threshold in successive images in order to determine the state of the eye (open or closed).

**Fig 1:**
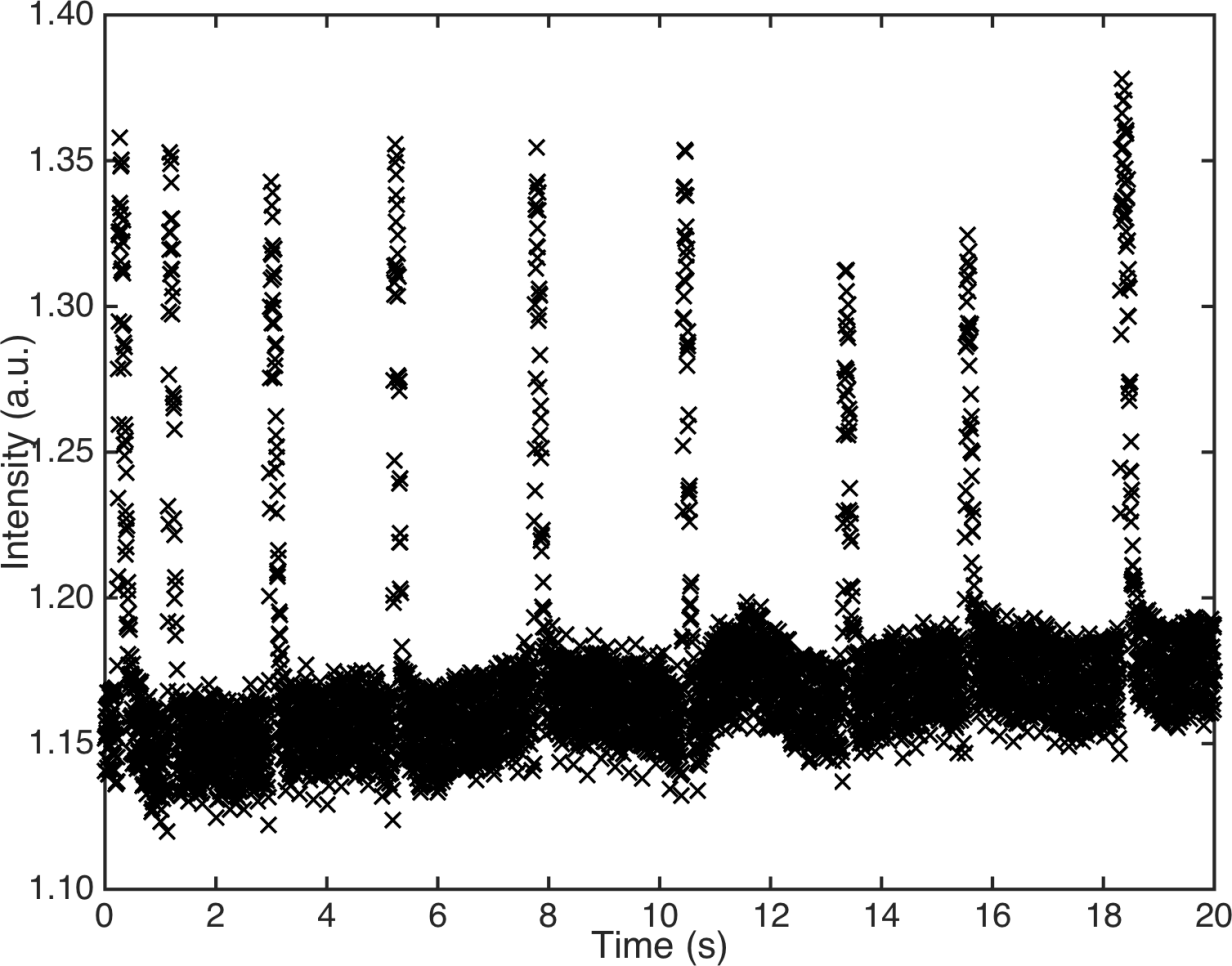
Reflected intensity vs. time. Variation in time of the sum of the intensity of the pixels computed for an example sequence. The intensity remains almost constant in time. Peaks correspond to the eyelid closed.

In this work, first, a rectangular ROI around each eye was selected. This was done by hand in the first frame of each sequence in order to make the algorithm computationally lighter, while in the following frames, the selection is automatic. The energy contained in each region was computed in all frames of all the sequences. The amount of light intensity reflected by the eye is almost constant when the eyelid is open. When the eyelid closes, the reflected light changes and so does the intensity that was registered by the camera. Therefore, blinks appear as fast increases and decreases of light intensity recorded by the camera. This variation is directly related to the variation of the eyelid position. Fig 1 shows an example of the variation in time of the sum of the intensity of the pixels of one ROI in a registered sequence.

In order to locate and isolate each blink from the sequence, we used a noise tolerant peak-finding algorithm. Peaks represent the instant when the eyelid is completely closed. Each peak is used as reference to extract blinks by cropping the sequence from 0.25 s (60 frames) before to 0.46 s (111 frames) after the peak maximum, which corresponds to the eyelid completely closed. The blinks with higher frequency that overlap in this interval were discarded. Previous works in literature measured a mean inter-blink interval of 5.97 s for normal versus 2.56 s for dry eye subjects [11] and defined the blinking as eyelid closures with a duration of 50 to 500 ms [18]. With the imposed limitations to the blink and inter-blink durations, we discarded incomplete and/or double blinks and consider all the range of closure duration for normal subjects.

Next, the curve of the intensity data (*I*_*i*_) versus time (*t*_*i*_) of each isolated blink was fitted using a smoothing spline *s*. The smoothing spline is the solution to the minimization problem shown in (1):

$$min\left\{p\;\sum_{i}{\left(I_{i}-s(t_{i})\right)}^{2}+(1-p)\int {\left(\frac{d^{2}s}{{dt}^{2}}\right)}^{2}dt\right\};$$ (1)

where *p* is the smoothing parameter set to 0.99996. The smoothed data are directly related to the position of the lid. Therefore, the approximated derivatives of *s*(*t*_*i*_) computed following (2) and (4) can be identified with the velocity, *v*(*t*_*i*_), and the acceleration of the lid, *a*(*t*_*i*_).

$$v(t_{i})=\left[s(t_{i+1})-s(t_{i})\right]/T\;;\;i=1,\ldots ,N-1;$$ (2)

$$\hat{v}(t_{i})=v(t_{i})/max(\left|v(t_{i})\right|);$$ (3)

$$a(t_{i})=\left[v(t_{i+1})-v(t_{i})\right]/T=\left[s(t_{i+2})-2s(t_{i+1})+s(t_{i})\right]/T^{2};$$ (4)

$$\hat{a}(t_{i})=a(t_{i})/max(\left|a(t_{i})\right|)$$ (5)

$\hat{v}(t_{i})$ and $\hat{a}(t_{i})$ respectively are the velocity and acceleration normalized to the maximum of their absolute value. *T* is the time interval between each one of the *N* samples of the data of intensity. In our case, *T* = 1/240 *s*, i.e. the inverse of the frame rate of the camera.

**Fig 2:**
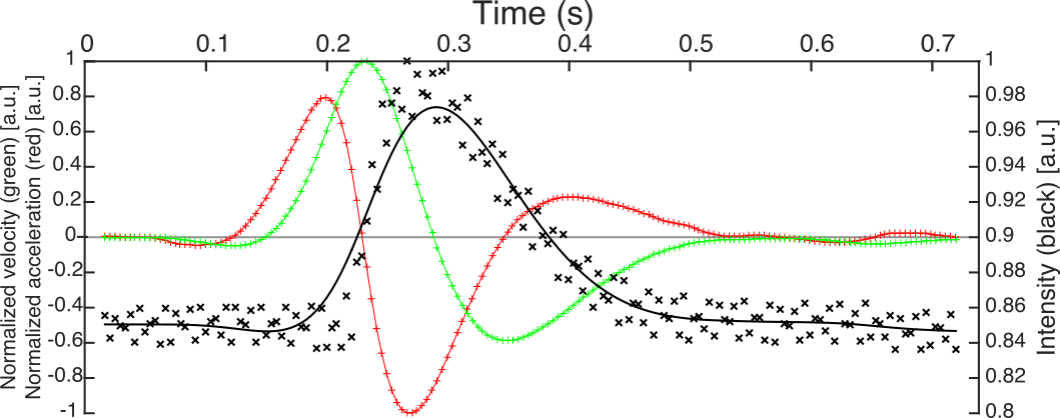
Normalized data of intensity, smoothing spline, velocity and acceleration for a blink. Data correspond to the first blink of the sequence of Fig 1. The data of intensity are the black crosses, the black line represents the smoothing spline, the data of velocity and acceleration are plotted in green and red lines, respectively.

In Fig 2, we represent the data corresponding to the first blink of the sequence presented in Fig 1. The data of intensity (black crosses) are normalized to the unity. Black line represents the smoothing spline *s*(*t*_*i*_) computed for the intensity following (1). The normalized velocity and acceleration are represented by the green and red lines, respectively.

Up to this point, although we have defined a temporal window to isolate each eye blink from the raw signal, the blink duration has still not been defined. As we stated at the Introduction section, the definition of the start and end of the phenomenon is approached in different ways in literature, however no one leads to a categorical criterion [30]. The start of the eye blink can be defined as the time when the velocity of the eyelid is zero before starting the displacement. However, this moment is difficult to define from the above computed curves. In Fig 2, one can see that both velocity and acceleration vary around zero before increasing their value while the position of the eyelid seems not to change.

The product of the velocity and the acceleration results in the power per unit of mass (4a). The power is the rate of doing work. It provides information about the work developed by a force, the lid muscles force, per unit of time in the blinking process.

$$P(t_{i})/m=v(t_{i})a(t_{i})=\left[s(t_{i+1})-s(t_{i})][s(t_{i+2})-2s(t_{i+1})+s(t_{i+2})\right]/T^{3};$$ (6)

$$\hat{P}(t_{i})=P(t_{i})/max(\left|P(t_{i})\right|).$$ (7)

**Fig 3:**
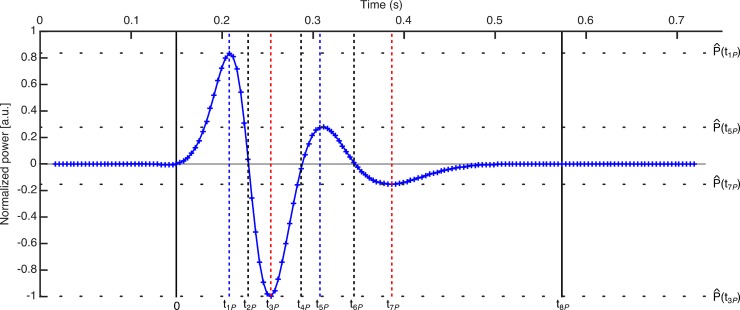
Normalized power per unit of mass developed by the eyelid muscles for the example blink. Black, blue and red dashed lines represent feature times obtained from zero-line intersections and local maxima and minima.

Fig 3 shows the normalized power, $\hat{P}(t_{i})$, computed following (7) for the example blink. When the eye is open (eyelid retracted), the sum of the activity of the muscles that take part in the blinking process is null. Muscles do not develop power so the power curve before and after the blink is zero. Therefore, the start and end of the blink can be defined just by determining when the curve respectively is different to zero and turns back to be zero. The normalized power can also be used to characterize other blink dynamic and kinematic features. Following with Fig 3, once the blinking has started, i.e. after the first vertical black line, the curve intersects the zero line three times during the blink process duration (black dashed vertical lines). These moments are when the velocity or the acceleration of the eyelid are zero. Additionally, the power curve shows two local maxima (blue dash lines) and minima (red dashed lines). The timing of the physiological process is as follows: a few hundredths of a second after the blink has started, the total power developed by the muscles is maximum at the time *t*_1*P*_ in the closure phase (1st blue-dashed line). Next, in *t*_2*P*_, the eyelid muscles stop working, the power turns zero and the eyelid gets a maximum velocity of closure (1st black-dashed line). After that, the eyelid starts braking and the power is developed with the opposite sign. There is a moment (*t*_3*P*_) when the curve reaches the minimum, which corresponds to the maximum power developed to brake the closure of the eyelid (1st red-dashed line). Then, the power decreases in absolute value until it returns to zero (2nd black-dashed line). This moment (*t*_4*P*_) corresponds to the eye closed, when the closure phase ends and the opening phase starts.

The shape of the curve in the opening phase is similar to that in the closure one. The total power developed by the muscles reaches a local maximum at *t*_5*P*_ (2nd blue-dashed line), which happens when the eye is in the upward phase. Then, the power diminishes until it is zero at *t*_6*P*_ and the eyelid reaches a maximum velocity (3rd black-dashed line). After that, the sign of the power changes when the eyelid opening is braking and the curve reaches a local minimum (2nd red-dashed line) at *t*_7*P*_. At that moment, the eye still is not completely open. Finally, the power decreases in absolute value until zero (*t*_8*P*_), when the eyelid is again retracted, the muscles forces are compensated and the blink is finished (2nd black line).

**Fig 4:**
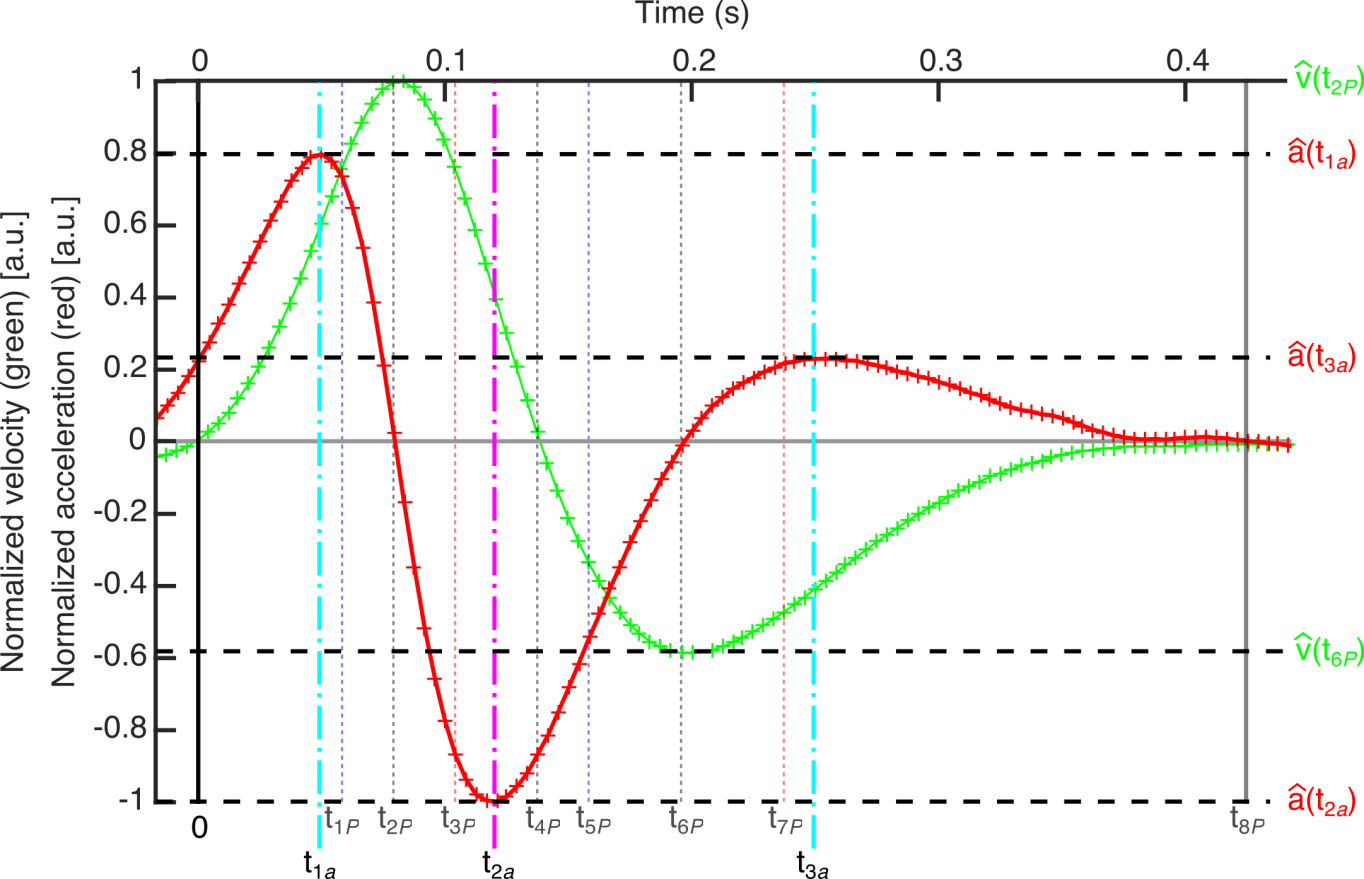
Normalized velocity and acceleration computed for the example blink. Velocity and acceleration are plotted in green and red lines, respectively. Local maxima and minima of the acceleration are used to define three new features (*t*_1*a*_, *t*_2*a*_ and *t*_3*a*_).

The values of the normalized power at the above described instants can be of interest to characterize the blinking. Therefore, we obtained the absolute values of the local maxima $\left|\hat{P}(t_{1P})\right|$ and $\left|\hat{P}(t_{5P})\right|$, and local minima, $\left|\hat{P}(t_{3P})\right|$ and $\left|\hat{P}(t_{7P})\right|$. Moreover, zeros of the acceleration are local maxima and minima of the velocity. However, the local maxima and minima of the acceleration do not match to those from the power per unit of mass. For example, in Fig 4, we represent the normalized velocity and acceleration computed for the above case with the time features previously obtained. We have shifted the time scale so that the blinking starts at time equal to zero. One can see that the zeros and the local maxima and minima of the velocity (green line) have already been characterized whereas local maxima and minima of the acceleration provide new time features.

Chronologically, *t*_1*a*_ is the time after beginning the blinking when the eyelid is in the closure phase and reaches a maximum in the acceleration. Next, after the maximum in the developed power and reaching a maximum in velocity, the total force brakes the eyelid (a change in the sign of the acceleration). This braking force reaches its maximum at *t*_2*a*_, before closing the eye. Then, in the opening phase, the dynamics is similar. The sum of forces accelerates the lid until a maximum at *t*_3*a*_. Later, that force diminishes and probably reaches a local minimum, which corresponds to the time when the eyelid’s braking acceleration in the upward phase is maximum, just before stopping the blinking. However, contrary to what happens in the power curve, this braking phase does not appear clear in the acceleration graphs, so, that local minimum of acceleration cannot be defined.

By proceeding with an analysis similar to that performed with the power, we obtained the absolute local peaks-values of normalized acceleration and velocity: $\left|\hat{a}(t_{1a})\right|$, $\left|\hat{a}(t_{2a})\right|$, $\left|\hat{a}(t_{3a})\right|$, $\left|\hat{v}(t_{2P})\right|$ and $\left|\hat{v}(t_{6P})\right|$.

**Fig 5:**
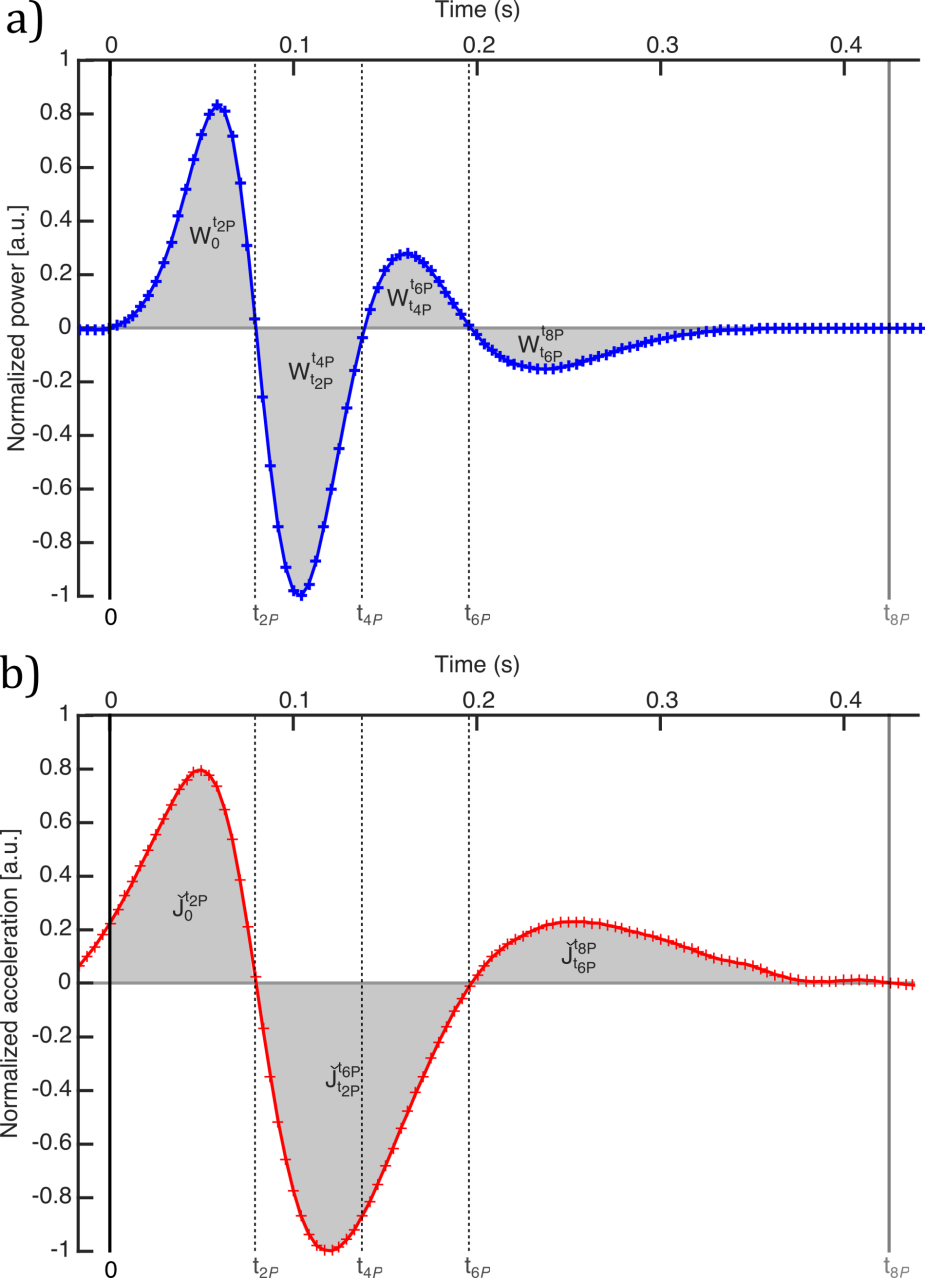
Normalized power per unit of mass and normalized acceleration. a) Normalized power per unit of mass. Gray areas represent the work developed by muscles between each zero-line intersection. b) Normalized acceleration. Gray areas represent the impulse of the eyelid at different stages.

Other magnitudes that we used to analyze the dynamics of the blinking were the work and the mechanical impulse developed by the eyelid muscles. The work done by those muscles is defined as the integral of the power developed by them (*P*(*t*) = *d W*(*t*)/*dt*)). Therefore, the area under the curve of the normalized power is related to the work developed by the muscles in a given period of time. Following the Eq (8), four new features are defined: $W_{0}^{t_{2P}}$ is related to the work performed from 0 to *t*_2*P*_, $W_{t_{2P}}^{t_{4P}}$, from *t*_2*P*_ to *t*_4*P*_, $W_{t_{4P}}^{t_{6P}}$, from *t*_4*P*_ to *t*_6*P*_ and $W_{t_{6P}}^{t_{8P}}$, from *t*_6*P*_ to *t*_8*P*_ (see Fig 5A).

$$W_{t_{c}}^{t_{d}}=T∙\sum_{t_{c}}^{t_{d}}\hat{P}(t_{i}).$$ (8)

Similarly, the area under the curve of the acceleration in a time interval is represented by J, the mechanical impulse per unit of mass developed by the muscles in that period of time:

$$J(t)=\int_{t_{c}}^{t_{d}}m∙a(t)dt;$$ (9)

$${\overset{ˇ}{J}}_{t_{c}}^{t_{d}}=J_{t_{c}}^{t_{d}}/\left[m∙max(\left|a(t_{i})\right|)\right]=T∙\sum_{t_{c}}^{t_{d}}\hat{a}(t_{i})$$ (10)

We have computed a magnitude proportional to the mechanical impulse developed by the eyelid muscles in different intervals following the Eq (10): ${\overset{ˇ}{J}}_{0}^{t_{2P}}$, from the start to *t*_2*P*_, ${\overset{ˇ}{J}}_{t_{2P}}^{t_{6P}}$, from *t*_2*P*_ to *t*_6*P*_, and ${\overset{ˇ}{J}}_{t_{6P}}^{t_{8P}}$, from *t*_6*P*_ to the end (see Fig 5B).

**Fig 6:**
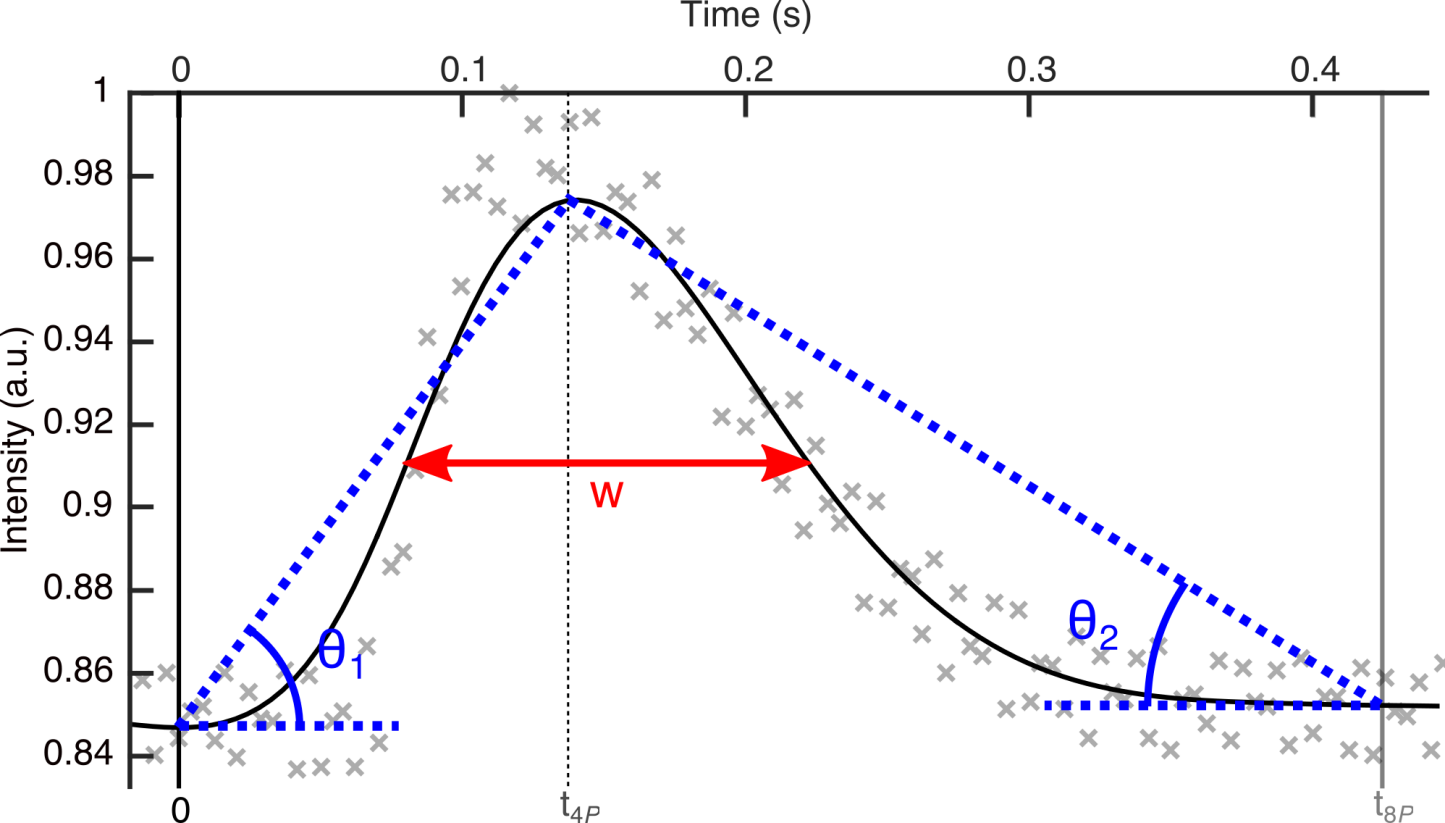
Displacement of the eyelid. θ_1_ and θ_2_ describe the mean velocities in the downward and upward phases, and w stands for the fwhm of the displacement.

Finally, from the analysis of the displacement curve *s*(*t*_*i*_), we have defined two more features that characterize the blinking: the full width at half maximum (fwhm) of the curve of the eyelid displacement in time, w, and the relation between the mean velocities in the closure and opening phases (*S*), given by Eq (11). In Fig 6, we represent both features for the blink that we are using as an example.

$$S=tan(\theta_{1})/tan(\theta_{2})=\left[\left(s(t_{4P})-s(t_{0}))(t_{8P}-t_{4P}\right)\right]/\left[(s(t_{4P})-s(t_{8P}))t_{4P}\right]$$ (11)

## Results

We applied the just explained analysis to the recorded video sequences and obtained 3251 eye blinks, ranging from 74 to 191 blinks per subject. The difference in the number of blinks per subject was due to losses in the processing of the signals (overlapping or incomplete blinks) and different subjects’ blink rates. However, all subjects retained at least 74 trials, so 74 random trials were selected from each participant to keep the size of each participant’s data set uniform (http://hdl.handle.net/10045/74398). Thus, we reduced the number of blinks resulting in a set of 1924 blinks (74 blinks for each of the 26 subjects). The above defined blink features were computed and grouped for each blink in a vector ${\vec{M}}_{j,k}=\{M_{j,k}(f)\}$, being j = 1,…,n subjects, k = 1,…,b trials and f = 1,…,F features, following the order stated in Table 1. There, we also present the resulting mean (12) and standard deviation (13) of all the features of the set of blinks under study.

$$\bar{M(f)}=(1/B)\sum_{j=1}^{n}\sum_{k=1}^{b}M_{j,k}(f);\;B=n∙b;$$ (12)

$$\sigma (f)=\sqrt{[1/\left(B-1\right)]\sum_{j=1}^{n}\sum_{k=1}^{b}{\left|M_{j,k}(f)-\bar{M(f)}\right|}^{2}};$$ (13)

**Table 1:** Features computed for the set of 1924 blinks.

*f*	$\bar{M(f)}$	*σ*(*f*)	Feature	Brief description^a^	
1	0.065	0.012	*t*_1*P*_	Time (s)	Local max. pow.
2	0.088	0.013	*t*_2*P*_		Zero pow. crossing
3	0.113	0.015	*t*_3*P*_		Local min. pow.
4	0.148	0.019	*t*_4*P*_		Closed
5	0.169	0.028	*t*_5*P*_		Local max. pow.
6	0.205	0.032	*t*_6*P*_		Zero pow. crossing
7	0.237	0.037	*t*_7*P*_		Local min. pow.
8	0.392	0.088	*t*_8*P*_		End (Zero pow.)
9	0.765	0.102	$\left|\hat{P}(t_{1P})\right|$	Normalized absolute power	
10	0.997	0.019	$\left|\hat{P}(t_{3P})\right|$		
11	0.415	0.180	$\left|\hat{P}(t_{5P})\right|$		
12	0.303	0.169	$\left|\hat{P}(t_{7P})\right|$		
13	7.407	1.124	$W_{0}^{t_{2P}}$	Work (a.u.)	From 0 to *t*_2*P*_
14	8.530	1.140	$W_{t_{2P}}^{t_{4P}}$		From *t*_2*P*_ to *t*_4*P*_
15	3.528	1.462	$W_{t_{4P}}^{t_{6P}}$		From *t*_4*P*_ to *t*_6*P*_
16	4.009	1.684	$W_{t_{6P}}^{t_{8P}}$		From *t*_6*P*_ to *t*_8*P*_
17	0.055	0.012	*t*_1*a*_	Time (s)	Max. acc.
18	0.131	0.017	*t*_2*a*_		Min. acc.
19	0.242	0.036	*t*_3*a*_		Max. acc.
20	0.703	0.141	$\left|\hat{a}(t_{1a})\right|$	Normalized absolute acc.	
21	0.997	0.021	$\left|\hat{a}(t_{2a})\right|$		
22	0.327	0.122	$\left|\hat{a}(t_{3a})\right|$		
23	14.040	1.254	${\overset{ˇ}{J}}_{0}^{t_{2P}}$	Impulse (a.u.)	From 0 to *t*_2*P*_
24	23.981	2.669	${\overset{ˇ}{J}}_{t_{2P}}^{t_{6P}}$		From *t*_2*P*_ to *t*_6*P*_
25	9.590	2.287	${\overset{ˇ}{J}}_{t_{6P}}^{t_{8P}}$		From *t*_6*P*_ to *t*_8*P*_
26	0.997	0.008	$\left|\hat{v}(t_{2P})\right|$	Normalized absolute velocity	
27	0.694	0.146	$\left|\hat{v}(t_{6P})\right|$		
28	0.142	0.031	w	Time (s) fwhm of displacement	
29	1.778	0.528	*S*	Ratio between average velocities	

Shaded cells correspond to the closing phase and no shaded cells to the opening one. 24th, 28th and 29th features comprise both phases and are shaded with a lighter gray.

^a^acc. = acceleration, max. = maximum; min. = minimum; pow. = power

From Table 1, we can see that the closure is faster than the opening. The eyelid is closed at around 0.15 s and expends around 0.25 s in the opening phase. Thus, the closure is almost twice as fast as the opening phase. This agrees with the work of Schelini et al. [44]. To blink, the nervous system turns off the tonically active LP and the OO muscle rapidly lowers the upper eyelid. To raise the eyelid again, the OO activity ceases and the LP activity, which consists of raising and holding the eyelid up, resumes [3,7]. Regarding the absolute value of the developed powers, those of the closure phase are larger than those of the opening phase. The same happens with the absolute values of the acceleration. That reveals differences in the mechanics of both processes. Those differences can be thoroughly evaluated from the analysis of the work features. In the closure phase, the muscles develop more work than in the opening one.

**Fig 7:**
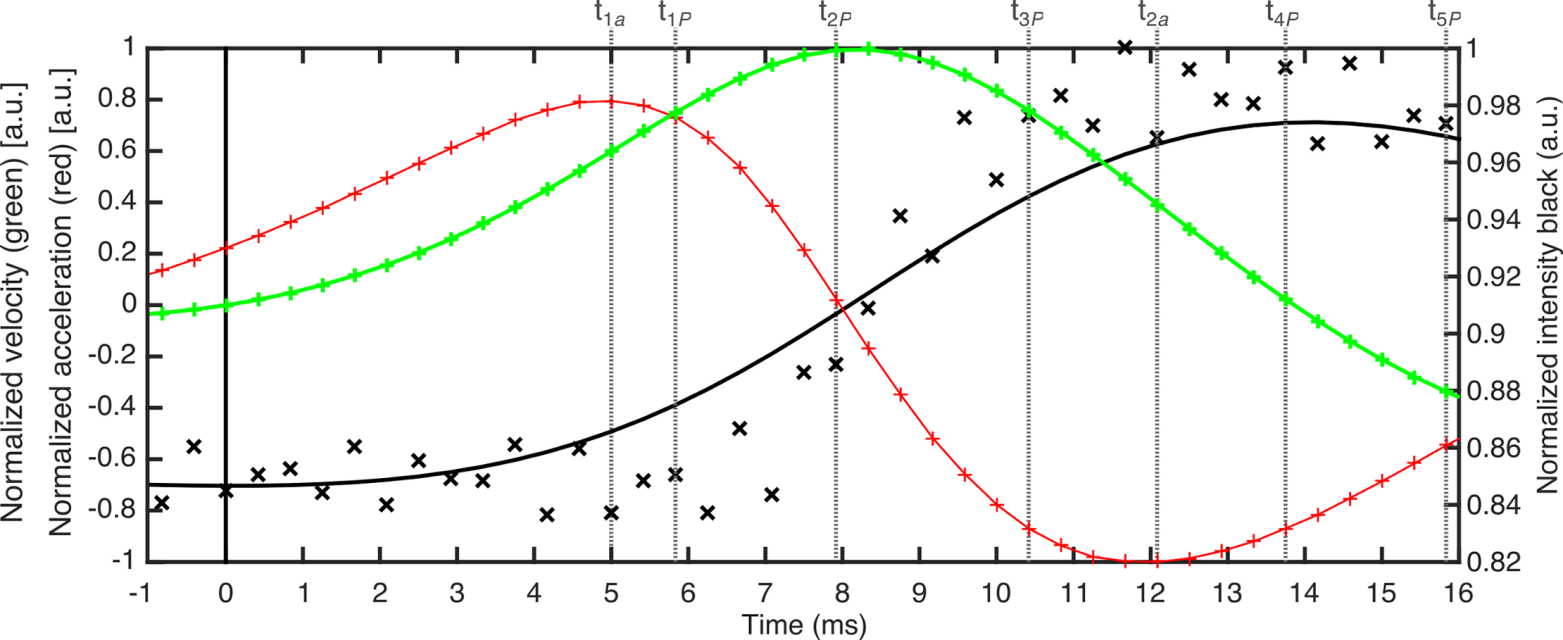
Eyelid displacement (black), normalized velocity (green) and acceleration (red) for the example blink.

The analysis of the maximum eyelid velocities both for closure and opening phase revealed that it was always faster in the closure than in the opening phase, in agreement with previous works [6,45]. Furthermore, Niida et al. [45] reported that, in the closure, the lid velocity shows two-phase distributions: an initial flat phase with small displacement and a second phase with a steep large displacement for the spontaneous blink. This fact is easily visible in the example in Fig 7. From the start to *t*_1*a*_ (the moment of maximum acceleration), along 5 ms, the displacement is small. Then, during the next 3 ms approximately and until the maximum closing velocity is reached at *t*_2*P*_, there is a large displacement (around half of the total distance to cover by the eyelid).

**Fig 8:**
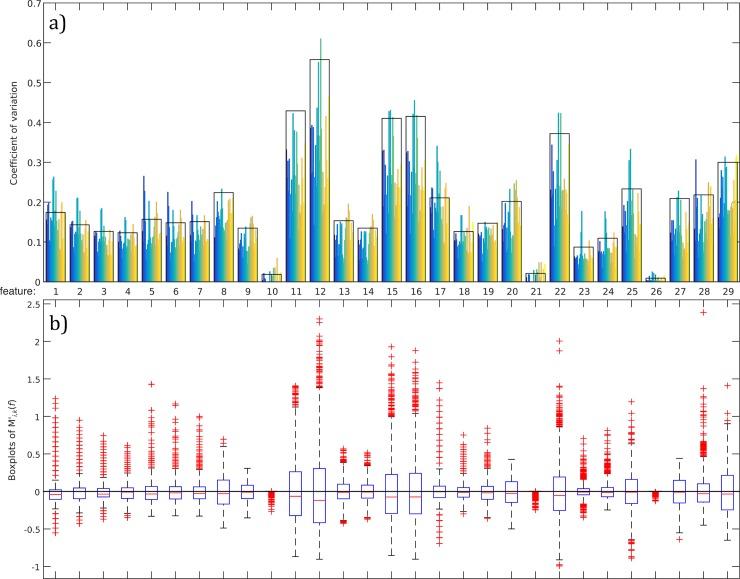
Coefficient of variation and boxplots of all the features. a) Coefficient of variation of all features for the blinks of each subject (color bars) and all the blinks (black bars). b) Boxplots of the features computed following Eq (9).

Eye blinking waveform can be used as biometric identifier, so features extracted from it does [20]. Thus, we propose using the obtained blinking features to biometric authentication. In order to test this possibility, we have first evaluated the discriminative ability of the extracted features. The coefficient of variation (CV), defined as standard deviation to the mean, permits assessing the inter and intra subject variability. A feature with a low intersubject CV means poor variability and therefore worthless discriminative skills. In Fig 8A), we have represented the CV of all the features computed for the 74 blinks of each subjects (blue to yellow bars) and for all the set of blinks (black bars). The graph manifests that the coefficient variation of the 10th, 21st and 26th features are always minimum (inter and intra subjects) and near zero. They respectively correspond to $\left|\hat{P}(t_{3P})\right|$, $\left|\hat{a}(t_{2a})\right|$ and $\left|\hat{v}(t_{2P})\right|$, i.e. the maximum absolute value of the power and acceleration braking the eyelid at the closure phase and the maximum velocity of the eyelid also at the closure phase. Their mean values in Table 1 are all close to 1, the maximum possible. They are maxima in around the 96% of all the measurements.

Another way to visualize the discriminative skills of the features is through boxplots. In Fig 8B), we represented the boxplots computed for all features following the Eq (14):

$${M^{\prime }}_{j,k}(f)=\left(M_{j,k}(f)-\bar{M(f)}\right)/\bar{M(f)}$$ (14)

Thus, $\bar{M(f)}$ values correspond to the zero line. Boxes are plotted in blue, the bottom and top of the box are always the first and third quartiles, and the red band inside the box is the second quartile (the median). The spacing between the different parts of the boxes indicate the degree of dispersion (spread) and skewness in the data. The narrower is the boxplot, the less scattered are the data. One can see that, for the 10th, 21st and 26th features, the upper and lower quartiles coincide with the mode and the average and there are no whiskers. Therefore, we discarded those features and did not use then in biometric classification.

Different classifiers like Linear and Quadratic Discriminant Analysis (LDA and QDA) [51,52], K-Nearest Neighbor (KNN) [53], Classification Tree (CT) [54] have been tested for the proposed system. A classifier assigns a new observation to a class from the training set. In our case, given a vector of features of a blink, any of the above classifier assigns that vector to a subject from the 26 subjects under study. The assignation can be the correct or not, so the performance of each classifier was evaluated through the correct identification rate (CIR), that is the correctly classified samples divided by the classified samples. The validation was performed through non-exhaustive cross-validation, concretely 10-fold cross-validation. Each set of data was proportionally partitioned into 10 disjoint subsets or folds. 9 folds were used for training and the last fold was used for evaluation. The process was repeated 10 times, leaving one different fold for evaluation each time.

We evaluated through 10-fold cross-validation the performance of the proposed features and the above classifiers over five sets of data: the original set with 1924 blinks (74 blinks for each of the 26 subjects) and four additional sets that were synthetically generated by using a bootstrapping procedure in a similar way to Armstrong et al. [21]. It consisted in generating 100 blinks for each participant. Each blink was constructed with the arithmetic mean of *β* random blinks of that participant’s 74 trials selected each time, being *β* = 3, 5, 10 and 25 for each set (named *β*-mean). This way, we constructed 100 unique vectors of *β* elements that range from 1 to 74. Each vector determined the *β* blinks from a subject used to compute each average blink. After this kind of resampling, 100 different blink features vectors were available from each participant, forming a set with a total of 2600 blink vectors for each *β* -mean set.

The different classifiers and sets can be compared through the computed CIR in Table 2. One can see that LDA provides better results than any other classifier with any set of data. We have validated this classifier using leave-one-out cross-validation, thus using one observation as the validation set and the remaining observations as the training set testing on all possible ways. Leave-one-out results agree with those obtained with 10-fold cross-validation so this non-exhaustive cross-validation method provide accurate results.

**Table 2:** Correct identification rate^A^.

Set	10-fold cross-validation				leave-one-out
	CT	QDA	KNN	LDA	LDA
Original	31.6	34.2	36.7	41.7	41.5
3-mean	57.9	56.7	64.7	75.7	76.5
5-mean	69.7	71.5	77.2	86.7	88.0
10-mean	84.3	85.4	91.1	96.0	96.5
25-mean	94.9	96.7	99.5	99.7	99.7

^A^Percentage obtained for all the classifiers and all the sets

The proposed method provided similar or better results than previous works. Thus, in the work of Abo-Zahhad et al. [20], with a training set composed by 50 blinks per subject from 25 subjects and taking as test averages of 25 blinks, the authors achieved a correct identification rate of around 97% in the best of the cases. Here, if we take averages of 25 blinks to construct the training set and bootstrap to get 100 blinks per subject, we reach a CIR for the LDA of 99.7%. For the 10-mean set and LDA classifier, the resulting CIR is a bit lower (96.5%) but taking the averages of only 10 blinks.

In an attempt to determine the discriminative skills of all features when they are used to biometric authentication, we have recursively tested the LDA algorithm. Starting from an empty feature set, candidate feature subsets were created by adding in each of the features that had not been yet selected. For each candidate feature subset, 10-fold cross-validation was performed by repeatedly calling the LDA algorithm with different training and test subsets. Each time the LDA was called, the number of misclassified observations was computed, i.e. the loss. Then, the candidate feature subset that minimized the loss was chosen. The process continued until adding more features did not decrease the loss.

**Fig 9:**
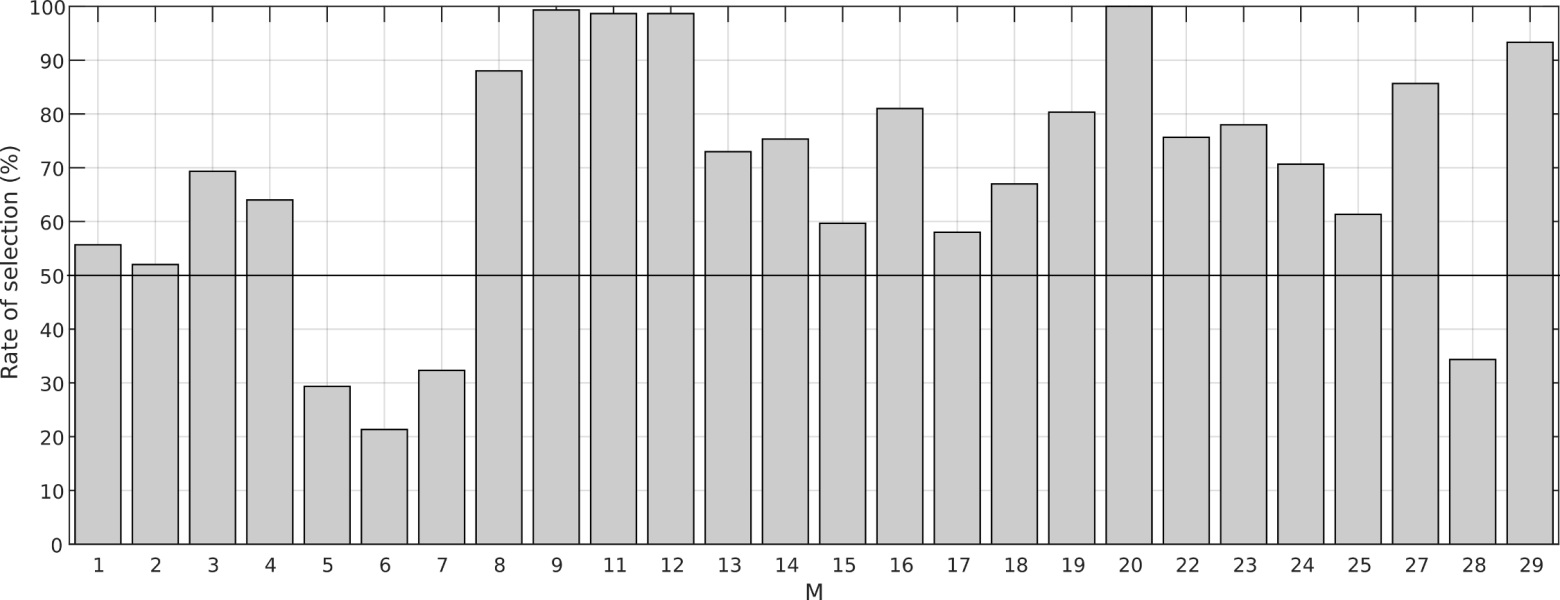
Rate of selection obtained for each feature. Percentage of selection of each blink feature obtained after recursively computing 60 times the feature subset that minimized the number of misclassified observations.

We recursively computed the features subsets selected for the trials subsets used above (original, 3-mean, 5-mean, 10-mean and 25-mean) 60 times. Thus, we got 300 vectors of selected features. In Fig 9, we represent the selection rate of the features. One can observe that almost all features are selected around half times at least, and only 3 of them (*f* = 5, 6 and 7) are selected around the 30% or less of times so they could be discarded as differentiating features. Contrary, some features are almost always selected. That mean that are good discriminant features. We could set a rate of selection threshold to define the best ones and use them in a new identification biometric system based on the combination of them with others from face recognition, fingerprint, iris, etc.

## Discussion and conclusions

We have applied different discriminant classifiers using different sets of vectors of blink features as an example of application of the presented method. Linear Discriminant Analysis provided the best correct identification rates for all the analyzed sets. We have constructed four different sets of blink vectors by computing new vectors from averages of vectors computed form real measurements. Resulting CIR improves with the number of samples used to compute the mean due to the fact that the intra-subject average vector eliminates noise but remains the discriminant ability of the features. Nevertheless, the 25-mean case may not be suitable for some practical application due to the fact that the identification process first would require obtaining at least 25 blinks from the subject. Taking into account the normal spontaneous interblink duration [55], that would imply recording blinking during more than 2 minutes. On the other hand, the system should be designed to authenticate using voluntary blinks. Then the recording time would drastically decrease. Anyway, the 10-mean case provided good results and the recording time is reduced to around 35 seconds.

Eye blinking is a physiological act related to intrinsic physical characteristics of the human body, so cannot be easily forged. This fact represents an advantage in its use to biometric authentication compared to others with lower security [20]. Moreover, our method is computationally less complex than authentication systems based on image processing of fingerprint, face or iris that deal with great number of data. Another advantage is that the authentication can be performed remotely and unconsciously for the subject. For instance, it can be used to identify or double-check a person in front of an ATM or a cell phone, or to prevent access of restricted contents in TV or computers. The use of blinking features to biometric authentication also may seem to present some drawbacks. On the one hand, it is well-known that fatigue is associated with increased blink frequency. Moreover, average individual rates of blinking increase with age [9,10] and those rates are correlated with dopamine levels in human and nonhuman primates [56]. Note that, in all cases, the blink feature that varies is the frequency or the blink duration in case of assessing fatigue. All the features presented in this work are obtained from one single blink, so the rate variation is not a problem. Of course, the variation of the blink latency will affect the performance of the biometric authentication and further analysis on this question should be done but it is out of our scope. On the other hand, the video sequences in this work are recorded in laboratory conditions, with approximately constant illumination and distance of capture. In real application, those conditions will probably vary and affect the performance of the method getting worse results. Fortunately, some solution could be applied to the registered signal in order to reduce the noise introduced by those changes. For example, in each frame, we could subtract the background light to the registered signal. It contains all variations due to changes in light or distance of capture. Background light could be estimated as the energy of a background region. Another possible solution would be employing Independent Component Analysis to the eye blink signal [57]. The validation of all these hypothesis, together with the evaluation of the authentication performance of the blink features combined with other biometric characteristics (finger-print, iris, face, …), remains as future work.

In this work, we have proposed obtaining distinctive subject features from a video sequence of the blinking taken with a widely available high-speed sports camera. We based our analysis on the fact that the change of the light reflected by the eyelid when it moves appears as changes in the registered intensity. The features extracted from the data describe the biomechanics of the blinking process and provide information about time, speed, acceleration, mechanical impulse, work and power developed by the muscles participant in the process. Up to our knowledge, kinematic parameters (position, speed, acceleration and the instants of time derived from them) are commonly used in the literature. However, the parameters related with the work, the impulse and the power developed by the muscles, and the times derived from them, are originally proposed in this work. Note that these include a categorical criterion to define the start and the end of the blink. Furthermore, results have shown that the power and the acceleration are maxima in absolute value when braking the eyelid at the closure phase and the maximum eyelid velocity is reached also at the closure phase. The method can be applied to deepen in the research of the blinking process and its relationship with fatigue, drowsiness, neurological diagnosis, etc. We have used the blinking features to biometric identification reaching a correct identification rate up to 99.7%.
